# Supplementary material for: A scoping review of the unmet needs of patients diagnosed with idiopathic pulmonary fibrosis (IPF)
Source: PLoS One. 2024 Feb 14;19(2):e0297832. doi: 10.1371/journal.pone.0297832 (PMC10866483; doi:10.1371/journal.pone.0297832)
Supplement: S1 Table — (PDF) [file pone.0297832.s001.pdf]

S1_ Table: Glossary

Unmet healthcare needs: The concept of unmet healthcare needs refers to difference between required and actually received health services in case of a health problem. (1) Examining these constructs is an important indicator to fully understand potential inequalities in health and can reflect imbalances that may exist at both the local and national level. (2) Availability of healthcare care focuses on the extent to which appropriate health services are available to meet the demands of those needing to access these services. (3) Accessibility represents the extent to which people can use health services and focuses primarily on the way that a person can get necessary medical services, reflecting constructs such as geographical location cost of services and social and cultural constructs such as language barriers.(1, 3)

Healthcare professional:

A health professional, also referred to as a healthcare professional or healthcare worker and occasionally abbreviated to HCP or HCW is a person who delivers care and services to the sick either directly or indirectly (4) People who may belong to this field include but is not limited to doctors, nurses, dietitian, physiotherapist, pharmacists, psychologist, occupational therapists.

Clinical nurse specialist:

Clinical nurse specialists (respiratory) provide expertise and specialist nursing services to patients with respiratory conditions. Respiratory CNS's roles are wide and varied and include and education, promotion of self-management, coordinating integrated care pathways and supporting the management of chronic respiratory disease. (5)

1. Levesque JF, Harris MF, Russell G. Patient-centred access to health care: conceptualising access at the interface of health systems and populations. Int J Equity Health. 2013;12:18.
2. Allin S, Grignon M, Le Grand J. Subjective unmet need and utilization of health care services in Canada: what are the equity implications? Soc Sci Med. 2010;70(3):465-72.
3. Mainz J, Worning A, Klazinga N, Gøtrik JK, Johansen KS. Policy on quality development for the medical profession. Ugeskrift for læger. 1992;154:3523-33.
4. Joseph B, Joseph M. The health of the healthcare workers. Indian J Occup Environ Med. 2016;20(2):71-2.
5. Rafferty S, Elborn S. The Role of the Respiratory Nurse Specialist. Clinical Pulmonary Medicine. 2004;11(4):228-36.
