# Supplementary material for: A scoping review of the unmet needs of patients diagnosed with idiopathic pulmonary fibrosis (IPF)
Source: PLoS One. 2024 Feb 14;19(2):e0297832. doi: 10.1371/journal.pone.0297832 (PMC10866483; doi:10.1371/journal.pone.0297832)
Supplement: S3 Table — (PDF) [file pone.0297832.s003.pdf]

S3_Table.pdf. Table search strategy on CINAHL

Search of CINAHL (EBSCO) conducted on 14th November 2022.

Search	Query	Records Retrieved
S1	(MH "Idiopathic Pulmonary Fibrosis") OR (MH "Idiopathic Interstitial Pneumonias+") OR (MH "Pulmonary Fibrosis+")	4,017
S2	TI (“Idiopathic pulmonary fibros*” OR “Idiopathic interstitial pneumonia*” OR “Familial Idiopathic Pulmonary Fibrosis*” OR “Usual Interstitial Pneumon*” OR “fibrosing interstitial lung disease” OR “progressive fibrosis” OR “nonspecific interstitial pneumonia” OR “pulmonary fibros*”) OR AB (“Idiopathic pulmonary fibros*” OR “Idiopathic interstitial pneumonia*” OR “Familial Idiopathic Pulmonary Fibrosis*” OR “Usual Interstitial Pneumon*” OR “fibrosing interstitial lung disease” OR “progressive fibrosis” OR “nonspecific interstitial pneumonia” OR “pulmonary fibros*”)	4,333
S3	TI ((service* OR need* OR support* OR care* OR caring OR nurs* OR pathway*) N4 (access* OR barrier* OR disparit* OR demand* OR gap	234,910
S4	(MH "Healthcare Disparities")	16,029
S5	(MH "Health Services Accessibility+") OR (MH "Health Services Needs and Demand+")	120,306
S6	S3 OR S4 OR S5	327,920
S7	S1 OR S2	5698
S8	S6 AND S7	100
