# Supplementary material for: A scoping review of the unmet needs of patients diagnosed with idiopathic pulmonary fibrosis (IPF)
Source: PLoS One. 2024 Feb 14;19(2):e0297832. doi: 10.1371/journal.pone.0297832 (PMC10866483; doi:10.1371/journal.pone.0297832)
Supplement: S4 Table — (PDF) [file pone.0297832.s004.pdf]

S4_Table.pdf. Data Extraction Instrument

**Evidence source details and characteristics**	
Author	
Year	
Title	
Country	
Aims/Purpose	
Study Population and sample size	
Concept	
Context or setting	
Study Design	
Methodology	
Patient needs identified (physical, psychological, other)	
PROMS Utilised	
Summary of key findings	
Limitations	
