# Supplementary material for: A scoping review of the unmet needs of patients diagnosed with idiopathic pulmonary fibrosis (IPF)
Source: PLoS One. 2024 Feb 14;19(2):e0297832. doi: 10.1371/journal.pone.0297832 (PMC10866483; doi:10.1371/journal.pone.0297832)
Supplement: S5 Table — (PDF) [file pone.0297832.s005.pdf]

S5_Table.pdf. Summary of findings from included information sources.

Reference Country	Aim	Sample size Recruit ment	Participants	Setting	% Male	Age range (mean) in years	ILD Type	Methodology	Summary of key findings
**Quantitative**									
1) Akiyama<br><br>2020<br><br>Japan	This study aimed to characterize the practice of pulmonologists regarding palliative care and end-of-life communication for patients with IPF and identify perceived difficulties and barriers to this.	135<br><br>Multi-site	HCP: Representative sample of pulmonologists from Shizuoka prefecture, Japan.	Pulmonologists working at up to 15 general hospitals in the region.	87.7% (total sample)	Age range between 20 and 60 with highest percentage (41.5%) in their 30's	IPF	Quantitative study utilising self-administered questionnaire s.	Findings reported that patients with IPF complained of dyspnea and cough. However, less morphine was prescribed for IPF then for lung cancer.<br><br>Participants experienced greater difficulty in providing palliative care for IPF patients then for lung cancer patients.<br><br>End of life discussions in patients with IPF were conducted later than the physician-perceived ideal timing.
2) Brereton<br><br>2020<br><br>UK & Ireland	The objective of this study was to investigate, within primary care healthcare systems, the time taken from the first primary care physician referral for symptom investigation to ILD specialist centre review and antifibrotic therapy commencement.	247<br><br>Multi-site	Patients diagnosed with IPF	Recruitment took place at two specialist ILD clinics in two countries with a primary care healthcare system (University Hospital Southampton, Southampton, UK, and Mater Misericordiae University Hospital, Dublin, Ireland)	74% (total sample)	Patients seen in 12 months or less (n-142) mean age 72 mean SD + 8.8 years. Patients seen in 12-24 months (n-41) mean age 73.5 SD + 7.39 years; patients seen after	IPF (N=247),	Quantitative study using electronic case note review.	Patients reviewed in the ILD clinic within 12 months of primary care referral had a significantly preserved mean forced vital capacity (FVC) (83±22% predicted) compared to those reviewed between 12 and 24 months (75±22%, p=0.036), and >24 months (71±17%, p⩽0.001), respectively.<br><br>Kaplan–Meier analysis identified that patients seen in the ILD clinic within 12 months from the initial primary care referral had significantly longer time to discontinuation of therapy compared to those seen at 12–24 or

						24 months (n-63) mean age 73.3 SD + 6.6 years.			>24 months.
3)Cove<br><br>2015<br><br>UK	The aim of this pilot study was to characterise the psychological needs of both patients and carers and to test the feasibility of a workshop approach.	29<br><br>Single site	13 patients with IPF 16 family members	Workshop	Male Patients: (n-10) Male Family members: (n-2)	unknown	Patients with IPF (n-13)	Quantitative questionnaire post workshop attendance	Preliminary data suggests attendance at a workshop addressing psychological issues is feasible and is perceived to be a positive experience by patients diagnosed with IPF and their families.
4) Dedent<br><br>2021<br><br>United States	Assess the geographic differences in mortality and baseline pulmonary function in participants with IPF.	849<br><br>Single site	Patients diagnosed with IPF.	UCSF ILD Clinic	Unknown	Unknown	IPF (n-849)	Retrospective cohort study Online data capture system.	Rural participants have decreased baseline pulmonary function and increased mortality within their first year of presentation to a tertiary care centre compared to suburban participants, independent of individual-level characteristics.
5)Johansson<br><br>2022<br><br>Canada	To test the hypothesis that greater travel distance to access ILD clinical care would be associated with more severe disease at time of referral and worse clinical outcomes.	1162<br><br>Multi-site	Patients with fibrotic Interstitial lung disease	Canadian Registry for Pulmonary Fibrosis (CARE-PF) which is a multicentre prospective registry of patients from ILD subspecialty clinics across Canada	49% (Total sample)	Mean age 63 ±12	Patients with fibrotic ILD (n=1162)	Quantitative study Online data capture system using registry data.	Patients with fibrotic ILD with a longer travel distance to their ILD clinic had better prognostic indices at baseline but had a higher risk of death or lung transplant in the total cohort and in patients with connective tissue disease related ILD.
6)Lancaster<br><br>2021<br><br>United states<br><br>Japan<br><br>France<br><br>Germany.	To gain physician and patient perspectives on the pathway to care from symptom recognition to diagnosis and disease burden.	Physicians (n=244) reported data on Patients with IPF (n=1249)	Healthcare professionals reporting on patients diagnosed with IPF plus a number of patients with IPF also self-reported (n=739)	Adelphi IPF II Disease Specific Programme, with the following countries included France, Germany, Japan and the United States.	68% (total sample)	Mean (SD) age at diagnosis of 65.6 years (SD 10.61)	IPF (1249)	Quantitative point-in-time study using an online survey.	Misdiagnosis and delayed diagnosis were common in this study; examples included a diagnostic delay of 0.8 years in Germany to 2.0 years in Japan after symptom onset was reported.<br><br>In all countries, patients more often reported symptoms in the survey than did their physicians.<br><br>On average, patients underwent 7–

		Multi-site							10 clinical tests before diagnosis.<br><br>Patients reported low quality of life with only 50% of patient with moderate/severe IPF were satisfied with their treatment.
7)Lamas<br><br>2011<br><br>USA	To examine the association between delayed access to subspeciality care and survival time in Idiopathic Pulmonary Fibrosis.	129<br><br>Single centre	Patients who met American Thoracic Society criteria for idiopathic pulmonary fibrosis	New York Presbyterian/Columbia University Medical Center (New York, NY)	76%	Mean age 63 years	IPF	Prospective cohort study using a questionnaire	The results of this single-centre prospective study demonstrate that delayed access to a tertiary care centre is associated with a higher rate of death from IPF independent of disease severity.
8)Lindell<br><br>2021<br><br>USA	To determine the feasibility, acceptability and efficacy of a nurse-led early palliative care intervention entitled “A Program of SUPPORT”, in patients with IPF and their caregivers.	76 dyads<br><br>Single centre	Patients with IPF (diagnosed in the year previous to their initial center visit)<br><br>Caregiver	University of Pittsburgh Dorothy P. and Richard P. Simmons Center for Interstitial Lung Disease at University of Pittsburgh Medical Center.	Patients’ intervention group: males, 80% Control group: males 85%	Patient group Age 65 (median age of 70 in intervention arm vs. 73 in control arm)	IPF and caregivers (n=76)	Randomized controlled trial.	Efficacy demonstrated a significant improvement in caregiver’s knowledge, disease preparedness, and confidence in caring for the patient as well as an improvement in knowledge and advance care planning completion in patient participants.
9)Maher<br><br>2017<br><br>United Kingdom	To investigate treatment patterns of European patients with IPF to understand antifibrotic prescribing and identify unmet needs in IPF treatment practice.	290<br><br>Multi-site	Respiratory physicians.	The questionnaire was developed by Elma Research, an independent market research agency, on behalf of F. Hoffmann-La Roche Ltd and distributed to physicians from France, Germany, Italy, Spain, and the UK.	Unknown	Unknown	IPF	Quantitative Study.<br><br>Patient chart review followed by an online questionnaire (35-40 min).	This audit highlights the high proportion of patient’s diagnosed with IPF, who do not receive approved antifibrotic treatment.<br><br>Overall, 54% of patients with IPF did not receive treatment with an approved antifibrotic. More patients had a confirmed IPF diagnosis in the treated (84%) versus the untreated (51%) population. Of patients with a confirmed diagnosis, 40% did not receive treatment. The treated population was younger than the untreated population (67 vs 70 years, respectively; $p \leq 0.01$), with more frequent multidisciplinary team

									evaluation (83% vs 57%, respectively; p ≤ 0.01). A higher proportion of untreated patients had forced vital capacity > 80% at diagnosis versus treated patients. Of patients with 'mild' IPF, 71% did not receive an approved antifibrotic versus 41% and 60% of patients with 'moderate' and 'severe' IPF, respectively.
10)Moor<br><br>2019<br><br>Denmark, Ireland, UK, Bulgaria, Czech Republic, Poland, Greece, Italy, Spain, Austria, Belgium, France, Germany and the Netherlands	The aim of the development of the joint expert and patient statement was to highlight the most pressing common unmet needs of patients with PF/IPF. Putting forward recommendations to improve the quality of life and health outcomes throughout the patient journey.	Patients (n=286) and HCP's (n=69)<br><br>Multi-site	Patient survey completed by: Patients 79%, Caregivers 21%<br><br>HCP 69:<br>56 physicians (81%) and 13 specialist nurses (19%).	Online survey distribution by the European Idiopathic Pulmonary Fibrosis and Related Disorders Federation (EU-IPFF) in 14 European countries.	70 % of the total patient group	Mean age 66 years of the patient group	Patients diagnosed with IPF (n=86%) Patients diagnosed with another type of PF (n=14%)	Quantitative study using online surveys.	Delays in diagnosis and timely access to interstitial lung disease specialist and pharmacological treatment were identified as important gaps in care.<br><br>Patients and HCPs reported that a greater focus on symptom-centred management, adequate information including trial information and increasing awareness of PF/IPF is required.
11)Moor<br><br>2021<br><br>Netherlands	The aim of this study was to evaluate the predictive value of the surprise question for 1-year mortality in IPF.	Questions answered by 140 HCPS in relation to 140 patients.<br><br>Single site	Patients with IPF (n=140)	A prospective cohort study at the Erasmus Medical Center.	87% (Total population)	Mean age 74 ±6.5	IPF (n=140)	Quantitative study Prospective cohort study using a paper questionnaire	The surprise question can accurately predict 1-year mortality in IPF using a multivariable model (OR 3.69; 95% CI 1.24–11.02; *p* = 0.019). The C-statistic of the surprise question to predict mortality was 0.75 (95% CI 0.66–0.85).
12)NCUBE	To create a nurse-led clinic to support, guide and optimise	Patients with IPF	Patients diagnosed with IPF.	Single site nurse led clinic.	Unknown	Unknown	IPF (n=9)	Creation of a nurse led	Needs amongst this small group varied from one patient who was

2020<br><br>New Zealand	therapy for patients with IPF.	(n-9)<br><br>Single site						clinic: including a quantitative pre/post clinic questionnaire	close to end of life and another patient who had just been diagnosed. Patient feedback at initial analysis was positive.
13)Personen<br><br>2018<br><br>Sweden	This cohort study was performed to explore potential differences in the care of IPF in two Nordic countries.	312 Finish registry (n-152) Swedish registry (n-160)<br><br>Multi-site	Finnish IPF Registry (n=152)<br>Swedish IPF registry (n=160)	Two national registries.	Finnish registry mean age (SD)<br>74.6 (8.3)<br>Swedish registry<br>72.5 (8.0)	Finnish registry 68.4%<br>Swedish registry 70.6%	IPF (n=312)	Quantitative study utilizing two patient registries using online data capture tool.	This study highlighted that there are differences in how patients are treated with antifibrotic drugs in Finland and Sweden.<br><br>To be resident in Sweden was the main determinant for receiving antifibrotic drugs (OR 5.48, 95%CI 2.65-11.33, P< 0.0001).
14)Sharp<br><br>2017<br><br>United Kingdom	To investigate the current practices around palliative and supportive care and to explore the impact of a supportive care decision aid tool.	73<br><br>Single site	The decision aid was prospectively assessed with 73 patients	UK ILD centre.	Pre-tool: 80.9%<br>Deceased: 82.8%<br>After-tool: 78.1%	Mean age<br>Pre-tool: 75<br>Deceased: 75.4<br>After-tool: 75	IPF	Quantitative study using a data capture tool.	The decision aid tool was completed for 49.3% of patients and resulted in significant increases in documented discussion of referral to palliative care (11.2% vs 53.6%, p<0.01).<br><br>Tool completion led to an increase in referral for palliative care (2.7% vs 16.7%, p<0.01).
15)Swaminathan<br><br>2022<br><br>USA	To identify clinical characteristics and social determinants of health that differentially associate with lung transplant compared with death in patients with IPF.	955<br><br>Multi-site	Patients diagnosed with IPF	IPF-PRO Registry (A multi-centre US registry of patients with IPF)	Male 75.5% (total sample)	Mean age 70 (65-75	IPF (N=955 total sample)	Quantitative study evaluating registry data.	For patients on the IPF-PRO Registry, median Zip code, income and access to a lung transplant centre differentially impact the risk of lung transplantation compared with death, irrespective of disease severity measures or other transplant eligibility factors.
16)Turnpenny<br><br>2015	This study aimed to explore chest clinicians' experiences in delivering care in advanced IPF.	57<br><br>Single site	Chest consultants (n=17), chest registrars (n=28), physiotherapists (11), and nurse clinician	Questionnaires were distributed at a regional respiratory meeting.	unknown	unknown	IPF	Quantitative study utilising questionnaire .	Less than 10% of all respondents felt they had significant training in initiating End-of-Life discussions or palliating symptoms.

UK			(n=1).						
17)Tyas<br><br>2019<br><br>UK	To explore if the introduction of severity criteria improved palliative care provision for patients with idiopathic pulmonary fibrosis.	47<br><br>Single site	Patients diagnosed with IPF.	Clinic setting	Unknown	Unknown	Clinic letter review of patients with IPF	Quantitative clinical audit tool.	Introducing IPF disease specific markers of severity, following the intervention from a SPCT consultant in 2016, along with having a respiratory consultant with a specialist interest in palliative care, has improved access to palliative care and symptom control for patients with IPF.
18)Van der Sar<br><br>2021<br><br>Europe	The aim of this study was to evaluate the time to diagnosis of pulmonary fibrosis, identify potential reasons for delays and document patients' emotions.	273<br><br>Multi-site	Patients with a self-reported diagnosis of pulmonary fibrosis.	EU-IPFF through its member patient organisations in Europe; these organisations distributed the survey to members and other patients through email and social media.	Unknown	Unknown	IPF (n=214) Sarcoidosis (n=28) Other types of PF (n=31)	Quantitative study utilizing surveys.	The time to diagnose pulmonary fibrosis varies widely across Europe.<br><br>Forty percent of individuals took ≥1 year to receive a final diagnosis.<br><br>Delays occur at each stage of the diagnostic pathway.
19)Wysham<br><br>2015<br><br>Sweden	To compare end of life care in oxygen-dependent Pulmonary Fibrosis patients compared to patients who died from cancer: A national population-based study.	Total: 57,128 patients with PF 285 & patients with terminal cancer 56,843<br><br>Multi-site	Patients with pulmonary fibrosis who died from January 1, 2011, through October 14, 2013 &<br>Patients who died from cancer in the same time period.	Nationwide Swedish Registries of long-term oxygen (LTOT) and EOL care: Swedevox and Swedish Registry of Palliative Care (SRPC)	PF 61% males Cancer patients 51%	Patients with PF 78 ±8 Cancer patients 75± 12	PF (N=285) Terminal cancer (n=56,843)	Quantitative study utilising nationwide Swedish registries.	PF patients access fewer palliative care services and experience greater symptom burden at the end of life than patients with terminal cancer.
20)Weatherald<br><br>2017	To describe the practice patterns of Canadian respirologists on the diagnosis	112 respondents to	Canadian Thoracic Society (CTS) members who are	Canadian Thoracic Society (CTS) member email	Unknown	Unknown	The majority of	Online quantitative surveys.	Practice patterns for diagnosis and treatment of IPF in Canada evolved in concordance with the publication of

Canada	and management of IPF and to determine if these patterns reflect current guidelines	the 2009 survey 107 respondents to the 2013 survey.<br><br>Multi-site	academic or community physicians, or respirology fellows-in-training were surveyed.	addresses used for survey distribution.			respondents were clinicians (86%)		international guidelines in 2011.<br><br>Access to palliative care, lung transplantation referral, multidisciplinary diagnosis discussion, and specialty IPF clinics were identified as gaps in IPF care in Canada.
**Mixed methods**									
21)Maher<br><br>2018<br><br>Canada, France, Germany, Italy, Spain, United Kingdom	The aim of this study was to investigate the views of patients with IPF and pulmonologists on the diagnosis and management of IPF to understand treatment patterns.	Physician group: (n-287) participated in the online questionnaire and (n-61) in the interview.<br><br>Patient group: (n-68) participated in interviews and (n-60) in online questionnaire.	Survey:<br>Patients with IPF (n=60)<br>Pulmonologists (n=287)<br><br>Interview<br>Patients (n=68)<br>Pulmonologists (n=61)	Patients recruited via physician referrals, patient groups, or market research panels.<br><br>HCPs recruited from market research panels.	72% (of total sample of patients)	Mean age of patients: 64.6 (±9.0)	IPF	Mixed method Interview (in person and telephone).<br><br>Online survey.	This study identified several barriers to antifibrotic treatment, principally reflecting the differing views and values of patient and physician were identified in this study suggesting a need for better patient -physician communication about pharmacological therapy for IPF.

		Multi-site							
22)Sampson<br><br>2015<br><br>UK	This study addresses the care needs of patients and carers at different stages of the IPF disease trajectory.	48<br><br><br>Multi-site	Patients diagnosed with IPF and paired carers.	Two UK specialist interstitial lung disease clinics	Patients: 18 males Carers: 6 males	Age range across the disease extent categories 56-87	Patients with IPF(n=27) Paired carers (n=21)	Cross sectional mixed methods study including interviews.	Patients diagnosed with IPF have a clear understanding of their prognosis but little understanding of how their disease will progress and how it will be managed.<br><br>Patients and carers outlined key elements of MDT activity capable of having significant impact on the care experience. They were structured around: focus on clinical encounters; timely identification of changes in health status and functional activity.; understanding of symptoms and medical interventions and coping strategies and carer roles.
23)Tikellis<br><br><br>2020<br><br><br><br>Australia	Report the experiences of people who participated in the Peer Connect Service and to document the resources required to support such a service.	32<br><br><br>Multi-site	Patients diagnosed with pulmonary fibrosis.	The Peer Connect Service managed by LFA, a national organization that provides support to people with a lung disease in Australia.	56%	Mean ±SD range 71± 7 (53–89)	32 peers consented to be interview ed.	Mixed methods study involving qualitative semi-structured interviews with Participants.	Major themes included the value of shared experiences, providing mutual support and the importance of shared personal characteristics (e.g. gender and hobbies) in allowing information and emotional support needs to be met.
24)Tikellis<br><br><br>2022<br><br><br>Australia	Examine the organization of IPF care across Australia, how it aligns with guidance for best practice, and identified barriers and facilitators to best care.	39<br><br><br>Multi-site	Purposive sampling of respiratory Physicians.	Hospitals and health networks with respiratory medicine services was identified through various sources that include the Australian Institute of Health and Welfare, the	unknown	unknown	IPF	Mixed Methods Online questionnaire and semi-structured telephone interviews.	Approaches to diagnosis, treatment and access to referral services were generally consistent with best practice guidance.<br><br>Barriers related to inadequate staffing, lack of a nurse coordinator, inadequate access to clinical trials and funding models.<br><br>Telehealth technologies were

				TSANZ website and internet searches.					perceived as facilitators to best care.
**Qualitative**									
25)Bonella<br><br>2016<br><br>Austria, Belgium, France, Germany, Ireland, Italy, Spain, UK, Netherlans.	The aim of this study was to gather perceptions from European patient advocacy groups regarding inequalities and unmet needs in IPF care, in order to develop a Patient Charter.	Patient advocacy group members interviewed from 11 PG's (n-12)<br><br>Working group (n-16)<br><br>Multi-site	Healthcare professionals and patient advocacy group members.	11 Patient advocacy groups	unknown	unknown	IPF	Qualitative interviews<br><br>Individual telephone interview using a .	Five key themes were identified, the need for improved diagnosis, treatment access, holistic care, disease awareness and palliative care.
26)Burnett<br><br>2019<br><br>Australia	The aim of this study was to assess the patient experience of modern IPF care.	100<br><br>Registry<br><br>Multi-site	Patients diagnosed with IPF.	The Australian IPF Registry,	61% (total sample)	Age range 57-90	IPF (N-100)	A Qualitative study using semi-structured interviews. (telephone).<br><br>Grounded theory.	Dissatisfaction with information received particularly at the time of diagnosis.<br><br>The burden of travel to specialist centres and costs of treatment were significant.
27)Cassidy<br><br>2021<br><br>Ireland.	Explore the palliative care and future health and social care planning requirements of individuals and families living with fibrotic interstitial lung disease (F-ILD) in Ireland.	60 delegates attended with 39 stakeholders particip	39 stakeholders participating in discussion: patients (n=12), caregivers (n=13), healthcare and adjunct professionals (n=9), industry representatives (n=4)	World Café setting.	Patients (n=5 male), caregivers (n=6 male)	Unknown	F-ILD	World-Café qualitative research approach using a constructivist paradigm.	Palliative care is fundamental to the care and treatment of F-ILD's, regardless of disease progression.<br><br>Unmet palliative care needs were identified as psychological and social support, disease education, inclusion of caregivers and practical and legal advice for disease progression and

		ating in discussi on.<br><br>Single site	and clergy member (n=1).						end-of-life planning.
28)Delameillieure<br><br>2021<br><br>Belgium	To understand the care trajectory for patients with IPF based on the perspectives of patients and healthcare professionals.	18<br><br>Single site	Healthcare professionals (n-9). Patients (n-9)	IPF disease management program at UZ Leuven.	Patient group (67%) and caregiver group (40%)	Mean age 70 years for patient group	Patient group (n-9) IPF	Qualitative study using interviews.	Overall findings identified pitfalls and suggestions for improvement covering all elements of the chronic care model (CCM), primarily at the level of the individual patient and the care team.<br><br>Self-management support.<br><br>Patient reported outcomes and eHealth tools.
29)Duck<br><br>2015<br><br>United Kingdom	To understand the perceptions, needs and experiences of patients with Idiopathic Pulmonary Fibrosis.	Patients (n-17)<br><br>Single site	Patients with a multidisciplinary team confirmed diagnosis of Idiopathic Pulmonary Fibrosis.	University Hospital of South Manchester Foundation Trust.	7 Males	Median age 67 years	IPF (N=17)	Qualitative study. Semi-Structured Interviews.	Three main themes were identified: ‘Struggling to get a diagnosis’; ‘Loss of the life I previously had’; and ‘Living with Idiopathic Pulmonary Fibrosis’. Patients reported struggling to get a diagnosis and coping with a life-limiting, rapidly progressive illness with no good treatment and few support structures.
30)Giot<br><br>2012<br><br>Switzerland Germany, France, Italy, Spain and the UK.	Researchers conducted a European survey of diagnosed IPF patients to identify unmet needs in the management of patients diagnosed with IPF and opportunities to improve care.	Patients (n=45) Carers (n=18)<br><br>Multi-site	Patients diagnosed with IPF and in some interview’s carers were also interviewed.	European wide survey of patients in Germany, France, Italy, Spain and the UK.	Unknown	Median age of patient’s was 67 years.	IPF (n=45)	Qualitative study utilizing structured interviews.	In 58% of cases, diagnosis was protracted due to dismissal of symptoms and misdiagnosis.<br><br>Patients treated in specialist centres reported better satisfaction with care than those treated by generalists.

									The patients cited negative impacts of IPF on almost all aspects of their lives, which could lead to depression.
31)Kalluri<br><br>2021<br><br>Canada	To explore the perspectives, experiences and needs of patients with IPF, family caregivers, and healthcare professionals on ACP-related experiences to understand and inform a framework for advance care planning.	20<br><br>Multi-site	HCP (N=10)<br>Patients (n=5) and caregivers (n=5).	Patients with IPF and family caregivers (PFCs) were recruited through the local Pulmonary Fibrosis Association. HCPs were recruited through email invitation letters to home care (HC) and acute care (AC).	Patients 60%<br><br>Caregivers 20%	Median age range patients: 69 (58-78)<br>Caregivers: 65 (20-73)	IPF (N=5)	Qualitative study using semi-structured interviews.	Participant perceptions included insufficient information and conversations occur late. Recommendations were to have earlier conversations; have open conversations; provide detailed information; and plan for end-of-life. Professionals related delayed timing to poor end-of-life care and distressing deaths.<br>Acute care professionals perceived lack of clarity of roles and described personal, patient and caregiver distress.
32)Lindell<br><br>2017<br><br>United States	Exploration of the perception of palliative care (PC) needs in patients with Idiopathic Pulmonary Fibrosis (IPF) and their family caregivers.	13<br><br>Single site	Convenience sample of patients (n=5) and family caregivers (n=5) & family caregivers of decedent IPF patients (n=3).	The University of Pittsburgh Dorothy P. and Richard P. Simmons Centre for Interstitial Lung Disease at UPMC, a multidisciplinary centre for ILD treatment.	Male patients 100%	Mean age of patient with IPF 71.4 ± 7.2	Time since diagnosis of IPF range 1-11 years	Qualitative study of patients and family caregivers using thematic analysis of focus group.	Four themes described 1) frustration with the diagnostic process and education received 2) overwhelming symptom burden, 3) hesitance to engage in advance care planning, and 4) comfort in receiving care from pulmonary speciality centre because of resources.
33)Masefield<br><br>2019<br><br>UK<br>Ireland<br>Belgium<br>Italy	The aim of this study was to identify the communication challenges reported by patients and carers across Europe and elicit ideas for improvement.	58<br><br>Multi-Site	Focus group participants: five patients and three carers in England; seven patients and four carers in Italy (two focus groups); six patients in Belgium; and twenty-three patients and ten carers in Ireland	Four national patient organisations in Europe held focus groups:	Unknown	Unknown	IPF	Qualitative focus group study.	Patients and carers in Europe have unmet communication needs, which could be met by specialist physicians and specialist centres providing more effective information and signposting to support services, including support groups and patient organisations.

			(five focus groups).						
34)Meadows<br><br>2017<br><br>USA	This study aimed to develop a new IPF patient/provider communication and disease education program.	16<br><br><br>Single site	Patients diagnosed with IPF (n-12) Caregivers (n=4).	The National Jewish Health (NJH) Interstitial Lung Disease (ILD) program	Patients (n=8 males) Caregivers (n=2 males)	Unknown	IPF (n=12)	Qualitative focus groups.	Patents desired information on improving their own quality of life and caregivers desired information on acquiring knowledge and skills they felt necessary to improve their efficacy as caregivers of a patient with IPF.<br><br>Patient and caregiver directed information should come in multiple formats.
35)Overgaard<br><br>2016<br><br><br>Denmark	The aim of this study was to increase knowledge of life with IPF for patients and family caregivers.	49<br><br><br><br>Multi-site.	Patients (n=25) Family Carers (n=24)	Specialist clinics at two University Hospitals	15 male participant s with IPF	Mean age Patients 71.1 (50-91)	IPF	Qualitative descriptive design using in-depth dyadic interviews with patients with IPF and family caregivers.	The following six themes were developed: information and disclosure, reactional dyssynchrony, perpetual vigilance, emotional ambivalence, gradual and tacit role shift, and adapted coping strategies.
36)Ramadurai<br><br>2018<br><br>USA	This study sought to improve understanding of the informational needs of patients and their caregivers.	17<br><br><br>Single site.	Patients diagnosed with IPF (n=13) Caregivers (n=4)	Interstitial Lung Disease Programme at National Jewish Health	Patients 54% Caregivers 50%	Mean age patients 68.1 (±7.2) Caregivers 63.3 (±7.7)	IPF	Qualitative study using focus groups.	Patients wanted information on how to live well despite having IPF, practical information on how they could remain active and travel and how they could preserve their quality of life despite living with a life-threatening disease like IPF.<br><br>Caregivers wanted information on the general aspects of IPF.
37)Russell<br><br><br>2016	The aim of survey was to probe the impact of IPF on patients' quality of life; the role of healthcare professionals and caregivers; the information	45	Multidisciplinary team-confirmed diagnosis of IPF with disease duration >3 months. Patient with	Patients were invited to enrol through patient support groups (UK), specialist	Male: 71%	Mean age 68.5	IPF	Semi-structured, qualitative, in-depth patient interviews of	87 % of patients reported that diagnosis took >1 year.<br><br>Patients reported that IPF had a significant physical and emotional

UK<br>Italy<br>Germany	needs of both patients and their caregivers; and patients' perceptions of pirfenidone as a new treatment option for IPF.	Multi-site.	IPF (n=45)	centres (Italy) or an advocacy group (Germany).				1-hour duration.	impact on their quality of life.<br>The beneficial role played by caregivers and interstitial lung disease specialist nurses (where available) was specifically highlighted.<br><br>Information was often of poor quality, out of date, or in English only.<br>Patients' perceptions of pirfenidone were largely positive and associated with 'hope' but were also influenced by the level of side effects experienced.
38)Schoenheit<br><br>2011<br><br>Germany, France, Italy, Spain, UK	To gain further insights regarding patients' perspectives on the diagnostic process, disease education, emotional well-being, and quality of life.	45<br><br>Multi-site.	Patients (20% from each country) with physician confirmed diagnosis of IPF.<br><br>Caregivers participated in 18 (40%) of interviews.	Germany, France, Italy, Spain, UK	49 % of patient sample	Median age: 67	IPF	Qualitative In-depth interview (in person).	The median reported time from initial presentation to confirmed diagnosis of IPF was 1.5 years (range 1 year between initial presentation and a confirmed diagnosis of IPF).<br><br>The most common unmet needs cited by participants were disease education resources, access to centres of excellence, and familial support programs.
**Literature Reviews**									
39)Bajwah<br><br>2017<br><br>United Kingdom	This review presents evidence for the epidemiology and management of the main symptoms of ILD and includes psychological needs and palliative care.	N/A	PF-ILD<br>ILD	N/A	N/A	N/A	ILD	Literature Review.	People living with ILD often suffer unmet physical and psychological needs throughout the disease journey.<br><br>Recent recommendations from the National Institute of Clinical Excellence promote the use of a new

									palliative care needs assessment tool.
40)Chaaban<br><br>2021<br><br>USA	Literature review to highlight the palliative care needs of patients with ILD.	N/A	ILD	N/A	N/A	N/A	ILD	Literature Review.	Palliative care addresses symptoms in diseases where cure is unlikely or impossible, especially chronic diseases.<br><br>Palliative care can, and should, be implemented early to addresses goals of patients and caregivers.
41)Kalluri<br><br>2019<br><br>Canada	This review sought to summarize the care pathway from the patient's perspective, identifying current gaps in care, education, support and communication among patients with IPF, their caregivers, and care teams during the patient journey.	N/A	N/A	N/A	N/A	N/A	IPF	Literature review.	The ability to assess and control symptoms; develop self-efficacy; access to psychosocial, emotional, and spiritual support; and information on the disease and its course are important unmet needs for both patients with idiopathic pulmonary fibrosis and caregivers.<br><br>There is a huge need to support the multidisciplinary work focused on identifying gaps across the patient journey and PREMS and PROMs can be instrumental to this goal.
42)Lee<br><br>2020<br><br>Australia	The supportive care needs of people with pulmonary fibrosis and their caregivers: A systematic review.	N/A	N/A	N/A	N/A	N/A	PF	Systematic Review.	A total of 35 studies were included in this review. The most frequently reported needs were in the domain of information/education and psychosocial/emotional needs<br><br>An additional domain 'access to care' was identified, this included access to peer support, psychological support, specialist centres and support for families of people with pulmonary fibrosis
43)Robalo-	Manuscript preparation which	N/A	N/A	N/A	N/A	N/A	N/A	Review.	Based on the available data from

Cordeiro<br><br>2017<br><br>Portugal	was the outcome of a multidisciplinary meeting between pulmonology, radiology, and pathology clinicians on the use of antifibrotic agents in IPF.								clinical trials and extension studies, the authors concluded that both Pirfenidone and Nintedanib have a significant effect on FVC decline.<br><br>Since IPF is a progressive lung disease, early diagnosis and treatment are crucial for slowing functional decline, reducing symptoms, and improving quality of life.
44) Van Manen<br><br>2017<br><br>Netherlands	Literature review to summarise the evidence regarding quality of life for patients with IPF and discusses challenges in the management of this devastating disease.	N/A	N/A	N/A	N/A	N/A	IPF	Literature Review.	The review summarises the most recent insights into measuring and improving quality of life for patients with IPF.<br><br>The authors propose a new model for continuous care in IPF – the 'ABCDE' of IPF care.
**Guideline/Policy document**									
45)EU-IPFF Report<br><br>2020<br><br>UK	EU-IPFF benchmarking report on access to Idiopathic Pulmonary Fibrosis (IPF) care in Europe	19 patient organisa tions involved in the study	Patient representatives and healthcare practitioners.	EU-IPFF member organisations and partners were invited to take part. There were 19 patient organisations from 17 EU Member States.	Unknown	Unknown	IPF	Qualitative study utilising phone interviews.	The Benchmarking Report outlines the current state of IPF care and management in Europe. It identifies best-performing countries along with challenges that require greater political attention and an immediate response.
46)HSE<br><br>2020<br><br>Ireland	National Framework for the Integrated Care Programme for the Prevention and Management of Chronic Disease in Ireland	N/A	N/A	N/A	N/A	N/A	Chronic disease	HSE National Framework.	This report sets out the national framework which will describe an integrated approach to the prevention and management of chronic disease in Ireland over the coming years (2020-2025).
47)Nice guideline<br><br>2017<br><br>United Kingdom	Idiopathic pulmonary fibrosis in adults: diagnosis and management	N/A	N/A	N/A	N/A	N/A	IPF	Guideline Document.	This guideline contains recommendations on the diagnosis of idiopathic pulmonary fibrosis and delivery of care to people with idiopathic pulmonary fibrosis.

48)The Irish Thoracic Society<br><br>2018<br><br>Ireland	Irish Thoracic Society Position Statement on the Management of idiopathic pulmonary fibrosis.	N/A	N/A	N/A	N/A	4/A	IPF	Position statement.	The standards of diagnosis and management of IPF need to be radically improved as outlined in the position statement.<br><br>All patients with IPF should have access to specialist centres and all appropriate treatments without undue delay
49)The Irish Thoracic Society<br><br>2018<br><br>Ireland	Respiratory Health of the Nation 2018.	N/A	N/A	N/A	N/A	N/A	Respiratory conditions	Review of Respiratory conditions in Ireland.	Future research direction: Research needs to be focused on people and patients to identify barriers in terms of diagnosis, self-care and access to health services.<br>Research is also needed to focus on quality-of-life outcomes as well as evaluation focused on implementation of interventions.
50)Irish Lung Fibrosis Association<br><br>2015<br><br>Ireland	National Patient Charter for Idiopathic Pulmonary Fibrosis	N/A	N/A	N/A	N/A	N/A	IPF	Patient Charter.	Six key patient entitlements outlined:<br>Early and accurate diagnosis.<br>Clear information about IPF.<br>Access to appropriate medicines and oxygen therapy.<br>Access to pulmonary rehabilitation and exercise programmes.<br>Early referral to the National Lung Transplant Unit for lung transplant assessment.<br>Access to psychological and palliative care services.
51)Matthews<br><br>2018<br><br>Ireland	Patient charter on dying, death and bereavement in Ireland.	N/A	N/A	N/A	N/A	N/A	N/A	Patient Charter.	The importance of reforming health and social care systems and wider societal structures to ensure that dying people have access to appropriate care and psychological and spiritual supports is being increasingly recognised.
52)Raghu	Idiopathic Pulmonary Fibrosis	N/A	N/A	N/A	N/A	N/A	IPF	Clinical	Recommendations for future

2022<br><br>United States	(an Update) and Progressive Pulmonary Fibrosis in Adults. An Official ATS/ERS/JRS/ALAT Clinical Practice Guideline							Practice Guidelines.	research include the following: optimize strategies for addressing quality of life, including treatment of comorbidities, physical activity, emotional well-being, and palliation of symptoms.
