# Supplementary material for: A scoping review of the unmet needs of patients diagnosed with idiopathic pulmonary fibrosis (IPF)
Source: PLoS One. 2024 Feb 14;19(2):e0297832. doi: 10.1371/journal.pone.0297832 (PMC10866483; doi:10.1371/journal.pone.0297832)
Supplement: Attachment — Submitted filename: Response to Reviewers.pdf [file pone.0297832.s007.pdf]

Dear Editorial team and reviewers,

I want to sincerely thank you for your time in reviewing our scoping review. Please find out comments below.

Reviewer has brought up an important point regarding certain biases related to abstract selection. Please address these comments and return for final approval. This will likely require that the co-authors clearly state w/in the methodology that North American abstracts were not reviewed, that the scoping review was limited to European sources, and should be noted consistently in the title and body. The other alternative is expand your review to include abstracts from North/South America and Asia. Please be clear and concise regarding the methodology and pool of applicable data that was utilized for the scoping review	Thank you for your comment. This list of conference abstracts was not intended to be exhaustive but rather a reflection of conferences abstracts that were reviewed as part of the systematic search of the literature as part of this extensive scoping review.<br><br>There were no geographic limits placed on the search strategy in this extensive review. In the first instance in the initial search of databases included in the search strategy abstracts were returned along with full text sources and the search was not limited to any particular geographic area.<br>In the second instance in the conference abstract search the American thoracic society abstract booklet was also reviewed as part of the scoping review.<br>We did not search conferences in Asia or South America specifically, but we are satisfied that based on the initial search which did not have geographic limits that we will have facilitated scope to include available literature including abstracts from those areas.
There is a contradiction between the exclusion criteria for posters (Table 4), which are presented as abstracts at scientific meetings, and the inclusion of abstracts as stated on page 9: "Several international meetings were reviewed, including the Irish Thoracic Society Annual Scientific Meeting. abstracts were reviewed, including those from the Irish Thoracic Society Annual Scientific Meeting, the European Respiratory Society Annual Meeting, and the British Thoracic Society meetings. A total of 100 abstracts were reviewed and 9 abstracts were selected for final review."	Thank you for your comments:<br><br>Table 4 now includes the following text: 'This review will not include case reports, protocols, letters, commentaries or opinion pieces'.<br><br>Page 9 now included the following text: 'Several international conference abstracts were reviewed including those from the Irish thoracic society annual scientific meeting, the European respiratory society annual meeting, the British thoracic society meetings and the American thoracic society meeting. A total of 100 abstracts were reviewed with 9 abstracts included in the final review'.
